# Two Decades of Cetacean Population Status and Mortality in Thailand: Spatiotemporal Trends, Environmental Drivers, and Anthropogenic Stressors

**DOI:** 10.3390/ani16111733

**Published:** 2026-06-04

**Authors:** Jindarha Prampramote, Worakan Boonhoh, Kannawee Swangneat, Chayanis Daochai, Watchara Sakornwimol, Orachun Hayakijkosol, Tuempong Wongtawan

**Affiliations:** 1 Marine Science Division, School of Science, Walailak University, Tha Sala, Nakhon Si Thammarat 80160, Thailand; jindarha.pr@wu.ac.th; 2 Marine Animal Research Initiative, Walailak University, Tha Sala, Nakhon Si Thammarat 80160, Thailand; worakan.bo@mail.wu.ac.th (W.B.); kannawee.sa@wu.ac.th (K.S.); 3 Akkhraratchakumari Veterinary College, Walailak University, Tha Sala, Nakhon Si Thammarat 80160, Thailand; 4 Centre for One Health, Walailak University, Tha Sala, Nakhon Si Thammarat 80160, Thailand; 5 Faculty of Veterinary Science, Prince of Songkla University, Hatyai, Songkhla 90110, Thailand; chayanis.d@psu.ac.th; 6 Marine and Coastal Resources Research Centre, The Central Gulf of Thailand, Department of Marine and Coastal Resources, Muang, Chumpon 86000, Thailand; watchavet70@gmail.com; 7 Veterinary Preclinical Science, Academy Division, College of Science and Engineering, James Cook University, Townsville, QLD 4811, Australia; orachun.hayakijkosol1@jcu.edu.au

**Keywords:** dolphin, whale, Andaman, Gulf of Thailand, climate

## Abstract

Cetacean mortality serves as a critical indicator of marine ecosystem health, reflecting the cumulative impacts of climate-driven environmental shifts and anthropogenic pressures. However, long-term national-scale assessments remain limited in Thailand. This study aimed to assess population status, analyse spatiotemporal mortality patterns, and evaluate the influence of environmental drivers and anthropogenic stressors in Thai waters over the past two decades. Secondary data from multiple sources were analysed using generalised linear models with a negative binomial distribution. A total of 29 cetacean species were recorded, with an estimated population of approximately 3000 individuals. Mortality was documented in 24 species and showed an increasing trend over time. Coastal species, particularly Irrawaddy dolphins and finless porpoises, accounted for the majority of deaths (56%). Mortality patterns varied significantly by region (*p* < 0.05) but not by season, with the highest levels observed in the Upper Gulf of Thailand. Environmental factors were significantly associated (*p* < 0.05) with mortality, including wind speed in the Andaman Sea and extreme conditions (drought and heavy rainfall) in the Upper Gulf. In the Lower Gulf of Thailand, mortality was significantly associated with a combination of environmental (sea surface temperature and wind speed) and anthropogenic factors (fishery production). Overall, environmental variability appeared to exert a stronger influence than anthropogenic stressors. These findings highlight the requirement for targeted monitoring in high-risk regions and periods, alongside improved investigation of mortality causes to support effective conservation strategies.

## Simple Summary

Whales, dolphins, and porpoises (cetaceans) are important indicators of ocean health, yet their mortality appears to be increasing. This study analysed 20 years (2005–2025) of cetacean status and mortality data in Thailand. Cetacean diversity in Thai water was high, with a total of 29 species recorded. Mortality showed an overall increasing trend, with coastal species—particularly the Irrawaddy dolphin and finless porpoise—accounting for most deaths. The highest death rate occurred in the Upper Gulf of Thailand. Environmental conditions were strongly associated with mortality, although their effects varied by region. Higher mortality was linked to strong winds in the Andaman Sea, extreme conditions in the Upper Gulf, and a combination of low temperature and strong winds in the Lower Gulf of Thailand. In contrast, human activities like vessel traffic and fisheries did not correlate with increased mortality. Overall, these findings suggest that changes in environmental conditions may play a major role in cetacean mortality in Thai waters, although multiple interacting factors are likely involved. Improving monitoring in key areas and times, and investigating additional causes of death, may support more effective conservation.

## 1. Introduction

### 1.1. Importance of Cetaceans

Cetaceans are of considerable ecological, economic, and conservation importance in Thailand, which lies between two highly productive marine systems—the Gulf of Thailand and the Andaman Sea—supporting a rich diversity of dolphin and whale species [1,2].

Ecologically, cetaceans function as ecosystem engineers that enhance marine productivity and stability. Baleen whales contribute to large-scale nutrient redistribution through the “whale pump” and “great whale conveyor belt”, facilitating the vertical and horizontal transport of nitrogen, phosphorus, and other elements across ocean basins [3]. Toothed whales and dolphins provide more localised nutrient subsidies; for example, sperm whales transport iron from deep waters to the surface, while coastal dolphins transfer nutrients between offshore foraging grounds and nearshore habitats such as coral reefs and seagrass beds [4,5]. Through these processes, cetaceans indirectly stimulate phytoplankton growth and enhance oceanic carbon sequestration, including long-term carbon storage following whale falls.

As apex predators and long-lived species, cetaceans also exert top-down control on marine food webs, preventing trophic imbalances and supporting biodiversity. Their longevity, high trophic position, and lipid-rich tissues make them effective sentinel species for monitoring bioaccumulated pollutants, harmful algal toxins, emerging pathogens, and antibiotic-resistant bacteria [6,7]. Consequently, cetacean health provides a valuable “One Health” indicator linking marine ecosystem integrity with human health risks.

Beyond their ecological roles, cetaceans contribute substantially to Thailand’s coastal economy through whale- and dolphin-watching tourism, consistent with the Blue Economy framework that promotes sustainable, non-extractive use of marine resources [8]. This transition from extractive activities, such as traditional fisheries, to service-based tourism recognises marine biodiversity as a natural capital capable of generating recurring economic value without resource depletion [9]. Given that the ocean economy accounts for approximately 30% of Thailand’s national GDP, cetacean-based tourism illustrates how living marine mammals can yield greater long-term economic returns than consumptive use [10].

Accordingly, documenting cetacean strandings and deaths serves as a practical framework for effective conservation, supporting not only biodiversity protection but also sustainable fisheries and long-term marine governance [3,5,6]. Under an integrated One Health paradigm, cetacean strandings and mortality are widely recognised as indicators of marine ecosystem health, providing valuable opportunities for pathological, toxicological, and epidemiological investigation through standardised necropsy protocols [11,12,13]. They reflect both natural processes (e.g., disease and prey shifts) and anthropogenic stressors (e.g., bycatch, vessel strikes, and pollution) [6,14].

However, while beach-cast stranding registries offer critical localised snapshots, comprehensive mortality data capture a broader spectrum of death events occurring further offshore or directly interacting with pelagic fleets, thereby yielding a more complete overview of regional mortality patterns. Nonetheless, the long-term interpretation of these stranding and death trends requires extreme caution; observed spatiotemporal patterns are heavily confounded by varying public reporting effort, carcass drift hydrodynamics, coastal human density, and environmental covariates such as monsoon wind velocities and coastal bathymetry [15]. Given that robust population abundance trends remain constrained by these observation biases, there is an urgent need to deploy integrated multi-stream monitoring approaches that combine stranding registries with systematic vessel surveys, passive acoustic monitoring, and photo-identification frameworks.

### 1.2. Current Status of Cetaceans in Thailand

Globally, cetacean conservation status has deteriorated over the past decade, with the proportion of threatened species increasing from 19% in 2008 to 26% in 2021 [12]. Anthropogenic pressures remain the primary drivers of mortality, particularly fisheries bycatch, which is estimated to cause approximately 300,000 deaths annually and disproportionately affects small cetaceans [13,15]. In contrast, ship strikes have emerged as a major threat to large cetaceans [16]. In addition to these direct pressures, environmental and climatic changes are increasingly recognised as important contributors to cetacean mortality. For example, thermal anomalies in enclosed basins have been linked to elevated mortality in small, isolated populations in the Amazon [17], while seasonal patterns such as the rainy season have been associated with increased mortality of endangered marine species in Thailand [18]. These environmental effects may be mediated through changes in oceanographic conditions, including reduced salinity. Indeed, prolonged exposure to low salinity has been strongly associated with dolphin mortality in the United States, likely through physiological disruption and increased susceptibility to disease [19].

Historical records documented 27 cetacean species in Thai waters [20], and ongoing long-term monitoring and molecular analyses continue to refine species inventories and distribution patterns. Recent surveys identify the Andaman Sea as a biodiversity hotspot, with 19 species from six families recorded between 2018 and 2023 [1]. Coastal species such as the Indo-Pacific humpback dolphin and the Irrawaddy dolphin are the most frequently observed, particularly around Phuket, Trang, and Surat Thani [1,21].

In Thailand, cetacean research evolved from simple stranding and death documentation to retrospective mortality analysis. Between 2018 and 2023, the Department of Marine and Coastal Resources (DMCR) recorded 231 cetacean strandings in the Andaman Sea, of which 82% were found dead [1]. Stranding and death frequency shows seasonal variation, with peaks during the monsoon period, likely influenced by environmental stressors and oceanographic conditions [18].

Although natural causes, such as disease, account for a substantial proportion of cetacean mortality in Thailand, anthropogenic impacts remain a significant concern [1,22]. Approximately 17.8% of examined carcasses exhibited signs of human-related trauma, primarily fishing gear entanglement [1]. Gillnet bycatch in small-scale fisheries is consistently identified as a major threat to coastal cetaceans worldwide, including in Thailand [23,24]. A recent study in Thailand suggests that dolphin-sightseeing tourism can generate noise pollution that alters the behaviour of coastal dolphins, potentially reflecting stress-related responses [24,25]. Routine vessel traffic has been shown to reduce the communication space of Bryde’s whales by up to 87–99%, depending on the distance between the whale and the vessel [26]. Furthermore, anthropogenic waste, particularly macroplastic debris, has been associated with cetacean mortality, especially in baleen species [27].

### 1.3. Gap in Knowledge

Despite more than two decades of cetacean stranding and death records in Thailand, comprehensive long-term analyses integrating population status, spatial patterns, temporal trends, and environmental and anthropogenic drivers remain limited. Existing studies have largely relied on short-term, regional, or opportunistic data, resulting in a fragmented understanding of nationwide patterns [1,25]. Most investigations have focused on species occurrence or descriptive mortality patterns, with limited quantitative evaluation of long-term spatiotemporal dynamics and the relative contributions of environmental and anthropogenic drivers [1,2,3,21,23].

Consequently, it remains unclear whether observed increases in strandings or mortality reflect true population changes, environmental variability, improved reporting effort, or increasing anthropogenic pressures. Moreover, the lack of integrated analyses combining environmental (e.g., monsoon cycles, sea surface temperature, oceanographic processes) and anthropogenic factors (e.g., fisheries interactions, vessel traffic, tourism pressure, and coastal development) has constrained the ability to disentangle their relative effects and identify regional hot spots. Consequently, a thorough, long-term spatiotemporal evaluation is essential to furnish evidence-based insights into cetacean mortality and facilitate conservation planning in Thai waters.

To address the fragmentation of historical national registries, this study established an integrated analytical framework combining multiple complementary data streams. Simultaneous evaluation of sighting records and stranding datasets was considered essential because each provides distinct but complementary ecological information. Systematic sighting records provide baseline information on species diversity, population distribution, occurrence boundaries, and demographic trends of resident cetacean populations, whereas long-term stranding and mortality records capture pathological anomalies and function as ecological sentinels of environmental change and localised anthropogenic stress. Integrating these datasets therefore enables a more comprehensive assessment of cetacean population status, mortality dynamics, and potential ecological drivers across Thai waters.

The specific objectives of this study were to: (1) assess cetacean species diversity, occurrence, and population status in Thai waters over a two-decade period; (2) investigate spatial and temporal patterns of cetacean strandings and mortality across the Upper Gulf of Thailand, Lower Gulf of Thailand, and Andaman Sea; and (3) evaluate the relative influence of environmental variability and anthropogenic stressors on regional mortality trends.

## 2. Materials and Methods

### 2.1. Ethics

No animal ethical approval was required due to the study being conducted using secondary data obtained from available reports.

### 2.2. Data Source

Secondary data on cetacean status and mortality were obtained from online citizen-based reports (e.g., https://www.thaiwhales.org), non-governmental organisation (NGO) records (e.g., https://www.seub.or.th/), news media sources (e.g., https://www.thaipbs.or.th/), and official records from the Department of Marine and Coastal Resources (DMCR; https://www.dmcr.go.th). Environmental data, including climatic variables, were acquired from the Thai Meteorological Department (https://www.tmd.go.th), while oceanographic data were sourced from the DMCR. Anthropogenic data were obtained from multiple agencies: tourist numbers from the Department of Tourism (https://www.dot.go.th/home), fishery production data from the Department of Fisheries (https://www4.fisheries.go.th/dof/main, accessed on 1 June 2026), information on coastal industrial estates from the Industrial Estate Authority of Thailand (https://www.ieat.go.th), and data on ports and vessel traffic from the Marine Department and the Port Authority of Thailand (https://www.ieat.go.th). Additionally, data on the number of tourist boats engaged in dolphin-watching activities was obtained from the Khao-ok ecotourism group, a local tour operator located at Nakhon Si Thammarat, the Lower Gulf of Thailand (https://www.khaook.com).

### 2.3. Sighting-Based Abundance Baseline and Population Estimation

Cetacean species recorded in Thai water were categorised into 2 broad ecological groups based on their predominant habitat use, foraging behaviour, and bathymetric distribution in Thai waters. “Coastal” (neritic) species primarily inhabit shallow, nearshore environments within the continental shelf and often exhibit site fidelity to coastal, estuarine, or lagoon environments, typically at depths < 200 m. In contrast, “oceanic” (pelagic) species comprise wide-ranging taxa typically associated with offshore and open-ocean environments beyond the continental shelf break, typically at depths > 200 m. These ecological classifications are descriptive and reflect generalised habitat preferences rather than strictly mutually exclusive biological boundaries.

Because systematic national abundance surveys are logistically constrained to nearshore environments, formal population estimation was restricted to six common resident coastal species: the Indo-Pacific bottlenose dolphin, finless porpoise, Indo-Pacific humpback dolphin, Irrawaddy dolphin, Bryde’s whale, and Omura’s whale. The data sourcing and modelling protocols for these species were divided by timeline and data stream availability. Annual abundance numbers for the period spanning 2017 to 2023 for five of the primary resident coastal species—excluding Omura’s whale—were retrieved directly from the long-term monitoring database maintained by the government (https://www.dmcr.go.th). For the cryptic Omura’s whale, for which formal government line-transect monitoring data are currently unavailable, annual presence and minimum population baselines were compiled from unconventional data streams, including verified online citizen-based reporting platforms (e.g., https://www.thaiwhales.org) and non-governmental organisation (NGO) logs (e.g., https://www.seub.or.th/). These integrated citizen science networks served as a vital spatiotemporal proxy for tracking this data-deficient species between 2017 and 2025.

Finally, because comprehensive empirical field surveys for the 2024–2025 period have not yet been published by state authorities, population figures for these two terminal years were mathematically projected. The projections utilised the empirical 2023 abundance peak as a baseline matrix. Future trajectories were modelled under a scenario-based demographic constraint framework, assuming a slight population decline following the 2023 peak. This reduction vector was calculated by subtracting the real-time annual regional mortality increments documented in our stranding database from the known baseline reproductive output limits of small cetaceans. Consequently, the 2024–2025 metrics should be strictly interpreted as scenario-based demographic estimates rather than field-verified absolute counts. Due to their highly transient nature, wide pelagic ranges, and the absence of dedicated baseline tracking frameworks within the Thai exclusive economic zone, broad oceanic species were excluded from absolute annual abundance counts.

### 2.4. Variable Definition and Classification

Variables included both categorical and continuous measures. Categorical variables comprised coastal region (the Upper Gulf of Thailand, the Lower Gulf of Thailand, and the Andaman Sea), season (land, monsoon, and tourist), and rainfall categories. Continuous variables comprised environmental metrics—including rainfall volume, wind speed, sea surface temperature, and salinity—and anthropogenic indicators. To account for localised pressures, region-specific anthropogenic proxies were utilised: vessel traffic intensity was examined exclusively for the Upper Gulf of Thailand, while the number of tourist boats was analysed for the Lower Gulf of Thailand. All continuous variables were averaged over the 2020–2025 study period.

The Upper Gulf of Thailand included the provinces of Samut Sakhon, Samut Songkhram, Phetchaburi, Prachuap Khiri Khan, Samut Prakan, Chachoengsao, Chonburi, Trat, Chanthaburi, Rayong, Bangkok, Chumphon, and Prachuap Khiri Khan. The Lower Gulf of Thailand comprised Surat Thani, Nakhon Si Thammarat, Phatthalung, Songkhla, Pattani, and Narathiwat. Although Phatthalung Province does not have a direct coastline to the open sea, it forms part of the Songkhla Lake lagoon system, a large brackish lagoon connected to the Gulf of Thailand, that supports a small resident population of Irrawaddy dolphins. The third region was the Andaman Sea, including Ranong, Phang Nga, Phuket, Krabi, and Satun.

Monsoon periods were categorised into 3 groups: Dry/inter-monsoon (Dec–Apr), Southwest monsoon (May–Sep), and Northeast monsoon (Oct–Nov). Land seasons were categorised into 3 periods: winter (Nov–Feb), summer (Mar–Jun), and rain (July–Oct).

Tourist seasons were categorised into 3 periods: high season (Nov–Feb), low season (May–Sep), and shoulder season (Mar–Apr, Oct). Continuous tourist numbers also included recordings from 2020 to 2025 from 3 provinces that represent each region and had the highest incidence of cetacean death in each region.

Rainfall volume was categorised into 4 groups: very small rain (<50 mm/month), small rain (50–100 mm/month), medium rain (100–200 mm), and heavy rain (>200 mm).

### 2.5. Statistical Analysis

The association between monthly cetacean mortality and potential factors was analysed using generalised linear models (GLMs). As the outcome (dependent) variable represented count data and exhibited overdispersion (variance greater than the mean), a negative binomial distribution was applied.

Explanatory variables were regional, environmental (climatic season, wind speed, sea surface temperature, and salinity), and anthropogenic (fishery production, vessel traffic, and tourism). In the GLM categorical variables were treated as factors, while continuous variables were included as covariates.

Associations were expressed as incidence rate ratios (IRRs) with 95% confidence intervals (CIs). Statistical significance was assessed using Wald χ^2^ tests, and post hoc pairwise comparisons between seasonal categories were performed using Bonferroni correction. Mortality estimates were derived from fitted negative binomial regression models. In univariable analyses, estimates were presented as estimated marginal means of mortality (EMM) for categorical variables and predicted mean mortality (PMM) for continuous variables. To illustrate the impact of continuous predictors, PMM was calculated at −1 standard deviation (SD) and +1 SD from the mean, representing values one standard deviation below and above the mean, respectively.

In multivariable analyses, adjusted EMM or PMM was reported as adjusted mortality, with all other covariates held constant. All analyses were conducted using Jamovi version 2.6.26 (open statistical software; https://www.jamovi.org) with package GAMLj3. Statistical significance was defined as *p* < 0.05, while *p* = 0.05–0.10 was interpreted as a marginal trend.

### 2.6. Statistical Justification for Model Specification

Because regional analyses were based on aggregated monthly data with a limited sample size (*n* = 12 months per region), multivariable models were restricted to a small number of predictors to avoid model overfitting and unstable parameter estimates. Variables with *p* < 0.20 in univariable analysis were considered for multivariable modelling to avoid excluding potentially important predictors, together with variables of biological relevance [28]. To minimise multicollinearity among correlated seasonal variables, only biologically distinct predictors (e.g., climatic and anthropogenic factors) were included simultaneously.

Continuous variables with large numerical ranges were rescaled (e.g., tourist numbers per 1000 individuals and fishery production per 1000 tonnes) to improve model stability and interpretability.

## 3. Results

### 3.1. Cetacean Species and Status in the Thai Water

Over the past 2 decades, 29 cetacean species from 6 families have been recorded in Thailand by government agencies, non-government organisations, and tourism operators. These include 6 coastal and 23 oceanic cetacean (Table 1).

All cetaceans are protected under Thailand’s Wild Animal Reservation and Protection Act B.E. 2562 (2019), administered by the Department of National Parks, Wildlife and Plant Conservation (DNP). Bryde’s whale and Omura’s whale are designated as Reserved Wild Animals, the highest level of legal protection under Thai law.

Under CITES, most large whales are listed in Appendix I, whereas most delphinids and beaked whales are included in Appendix II (Table 1). According to the IUCN Red List, two species are classified as Endangered (the Irrawaddy dolphin and the blue whale), four as Vulnerable (the finless porpoise, Indo-Pacific humpback dolphin, fin whale, and sperm whale), two as Near Threatened, and four as Data Deficient, while the remaining species are listed as Least Concern.

At the national level (Thailand IUCN assessment), all six coastal resident species are classified as Endangered, indicating greater conservation concern locally than globally. In contrast, most oceanic species have not yet been formally evaluated nationally (Table 1).

### 3.2. Baseline and Estimated Abundance of Resident Cetaceans

The estimated annual abundance of the 6 resident coastal cetacean species monitored in Thai waters between 2017 and 2025 exhibited a progressive increase, rising from 1554 individuals in 2017 to an empirical peak of 3002 individuals in 2023, thereby indicating an overall upward trend during the primary survey period. Following the 2023 peak, the demographic projections for 2024 and 2025 indicated a slight, subsequent stabilisation phase, with total numbers decreasing marginally to 2850 and 2725 individuals, respectively. Despite this minor scenario-based downturn, population levels remained substantially higher than the abundance baselines recorded prior to 2020 (Table S1).

Among the 6 assessed coastal species, the Irrawaddy dolphin consistently comprised the largest absolute proportion of the total resident population across all survey years, followed sequentially by the finless porpoise and the Indo-Pacific humpback dolphin. Conversely, the documented abundance of Bryde’s whales remained consistently low, while the cryptic Omura’s whale accounted for the smallest overall fraction of the aggregated population baseline (Table S1).

Furthermore, while the general coastal trends indicate regional population stability, specific isolated sub-populations remain under acute risk of localised extirpation. The critically small resident population of Irrawaddy dolphins in the semi-enclosed, brackish waters of Songkhla Lake demonstrated a severe, uninterrupted decline, dropping from approximately 100 individuals in 1995 to 30 in 2017, and further collapsing to an estimated 14 individuals by 2025, signalling an imminent risk of local extinction.

Irrawaddy dolphins also historically inhabited the lower Mekong River, including stretches along the Thai–Lao border; however, the population decreased from approximately 200–300 individuals in the early 1990s to 120–150 by the early 2000s and continued to decline until disappearing from the Thai–Lao section around 2022.

### 3.3. Spatiotemporal Trends in National Cetacean Mortality

The annual number of cetacean deaths during the study period. Mortality was very low in the early years (2007–2012), remaining below 10 animals per year. From 2014 onwards, the number of deaths increased substantially, with several peaks exceeding 35–40 animals between 2015 and 2019. A marked decline occurred in 2021–2022, when deaths decreased to approximately 13 animals. Following this reduction, mortality rose again, reaching the highest recorded value of just over 50 animals in 2025. The linear trend line indicates an overall increasing trend in cetacean mortality across the entire period.

**Figure 1:**
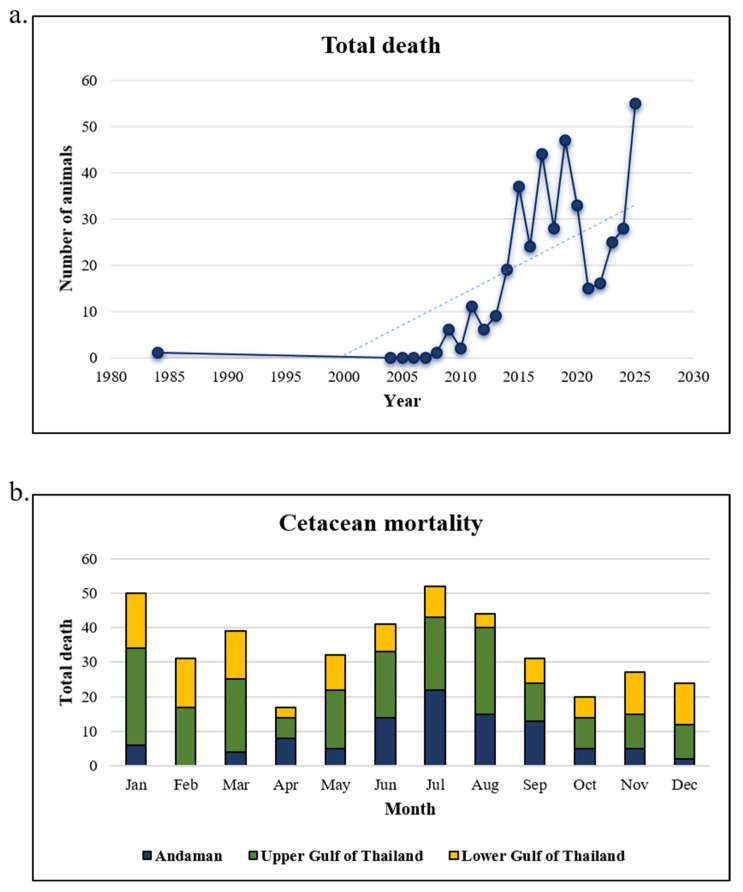
Temporal trends in cetacean mortality at annual (2005–2025) (**a**) and monthly scales (**b**). Numbers indicate the total number of cetacean deaths.

Monthly cetacean mortality showed clear temporal variation across the three marine regions of Thailand (Figure 1). Overall mortality was highest during the mid-year period, with peaks observed in July and August. The Upper Gulf of Thailand consistently recorded the highest number of deaths, particularly during July (21 deaths) and August (25 deaths). The Lower Gulf of Thailand showed relatively high mortality early in the year, with the highest monthly count in January (16 deaths), followed by moderate values throughout the remaining months. In the Andaman Sea, mortality was generally lower than in the Gulf regions but increased during the southwest monsoon period, peaking in July (22 deaths) and remaining relatively high in August (15 deaths) and September (13 deaths). In contrast, mortality in all regions tended to be lower during April and October (Figure 1). Overall, the results indicate regional and seasonal variation in cetacean mortality.

Between 2005 and 2025, a total of 408 cetacean deaths representing 24 species were recorded in Thailand (Table 1), of which 38 individuals could not be identified to species level. Conversely, 5 species have not been reported dead, including the ginkgo-toothed beaked whale, pygmy sperm whale, fin whale, blue whale, and humpback whale.

Among the 370 individuals identified to the species level, the highest number of deaths was recorded for the Irrawaddy dolphin (37.30%, *n* = 137), followed by the finless porpoise (18.38%, *n* = 68), Indo-Pacific humpback dolphin (8.92%, *n* = 33), and Indo-Pacific bottlenose dolphin (8.11%, *n* = 30) (Table 1).

Marked spatial variations in species-specific mortality were observed across the three marine regions. The striped dolphin was the most frequently reported dead species in the Andaman Sea (29.76%, *n* = 25), whereas the Irrawaddy dolphin was the most frequently reported species in both the Upper Gulf of Thailand (49.72%, *n* = 89) and the Lower Gulf of Thailand (33.64%, *n* = 36) (Table S2).

When aggregated by habitat-based ecological guilds, mortality was overwhelmingly concentrated among nearshore coastal residents, which accounted for 80.54% (*n* = 298) of all identified deaths. Pelagic oceanic delphinids accounted for the second-highest mortality load at 15.94% (*n* = 59), while deep-diving odontocetes (*n* = 11, 2.97%) and migratory oceanic baleen whales (*n* = 1, 0.27%) constituted the remaining documented losses.

### 3.4. National Analysis of Cetacean Mortality and Associated Factors

Using univariable analysis, associated factors for national cetacean mortality are shown in Table S3. Most factors were not significantly associated with cetacean mortality (*p* ≥ 0.1). Only regional (*p* = 0.004) and tourist factors (*p* = 0.001) were significantly associated with cetacean mortality, while rainfall was shown to have a marginal association (*p* = 0.054).

Regionally, the Upper Gulf of Thailand recorded the highest number of cetacean deaths (*n* = 194), followed by the Lower Gulf of Thailand (*n* = 115) and the Andaman Sea (*n* = 99). A significant regional effect on cetacean mortality was identified (χ^2^ = 10.89, df = 2, *p* = 0.004) (Table S3). Using the Andaman Sea as the reference category, the Upper Gulf of Thailand exhibited significantly higher mortality, with EMM of 16.17 deaths/month, corresponding to nearly double the incidence rate (IRR = 1.96, *p* = 0.002) (Table 2). In contrast, no significant difference was observed between the Lower Gulf and the Andaman Sea (*p* = 0.512) (Table 2).

Among environmental drivers, only rainfall volume showed a marginal positive association with cetacean mortality (χ^2^ = 3.70, df = 1, *p* = 0.054) (Table S3). Mortality increased from 9.36 deaths per month at lower rainfall levels to 13.25 deaths per month at higher rainfall levels. The incidence rate ratio (IRR = 1.00) indicates a slight increase in mortality per 1 mm increase in rainfall, although this effect was not statistically significant (*p* = 0.175) (Table S3). For anthropogenic variables examined, only tourist numbers were significantly negatively associated with cetacean mortality (χ^2^ = 9.99, df = 1, *p* = 0.001; Table S3). Cetacean mortality declined substantially with increasing tourist numbers, decreasing from 14.79 deaths per month at low tourist levels to 7.97 deaths/month at high tourist levels. For each increase of 1000 tourists, the expected mortality significantly decreases by approximately 1% (IRR = 0.99, *p* = 0.001).

The final multivariable negative binomial regression model included variables that were both statistically significant and biologically meaningful, namely region (the strongest predictor), rainfall (representing an environmental driver), and tourist numbers (representing an anthropogenic stressor). The overall model was statistically significant (χ^2^ = 19.80, df = 4, *p* < 0.001). Among the predictors, region and tourist numbers were significantly associated with cetacean mortality (*p* ≤ 0.008), whereas rainfall was not significant (*p* = 0.757) (Table 3).

Regionally, the Andaman Sea exhibited the highest adjusted mortality (56.08 deaths/month), compared with substantially lower levels in the Upper Gulf of Thailand (4.13 deaths/month; IRR = 0.07, *p* = 0.020) and the Lower Gulf of Thailand (4.86 deaths/month; IRR = 0.09, *p* = 0.008) (Table 3). Notably, this pattern differs from the univariable analysis, in which the Upper Gulf of Thailand showed the highest crude mortality, as adjusted mortality values represent model-based predictions with other covariates held constant.

Tourist numbers were significantly negatively associated with mortality (*p* = 0.002), with adjusted mortality decreasing from 47.33 deaths/month at low levels to 2.28 deaths/month at high levels (IRR = 0.99, *p* = 0.003), indicating a slight reduction in mortality with increasing tourism (Table 3).

### 3.5. Comparative Analysis of Crude Totals and Adjusted Multivariable Models

A critical analytical distinction must be maintained between raw descriptive regional totals and adjusted multivariable model outputs, as unadjusted temporal trends are heavily subject to environmental and anthropogenic confounding. In terms of crude, unadjusted descriptive patterns, the Upper Gulf of Thailand recorded the highest absolute mortality load (*n* = 194) across the two-decade study period, presenting a significant univariable regional effect (*p* = 0.002) that roughly doubled the incidence rate of the reference region.

However, upon implementing the final multivariable negative binomial regression model—which simultaneously controls for localised precipitation variations and tourism volumes—this descriptive pattern reverses. When all other covariates were held constant, the Andaman Sea yielded the highest adjusted predicted mortality baseline (56.08 deaths/month), whereas the predicted outputs for the Upper Gulf (4.13 deaths/month) and Lower Gulf (4.86 deaths/month) dropped significantly (*p* ≤ 0.020) below the reference baseline.

This statistical divergence shows that raw datasets are heavily influenced by local conditions that affect how easily carcasses are found and reported. In the Andaman Sea, heavy tourism occurs during calm weather, which naturally lowers the number of washed-up carcasses and hides the true environmental hazards. In contrast, the high raw numbers in the Upper Gulf are inflated by dense coastal populations and extreme weather anomalies that make carcasses much easier to see and report. Controlling for these factors removes local reporting biases, proving that the Andaman Sea actually carries a higher underlying baseline risk than raw numbers show. In practice, this means monitoring programmes cannot rely on raw stranding counts alone to identify risk areas; instead, management assets must be consistently deployed based on model-adjusted risks to protect populations in open marine environments.

### 3.6. Distribution of Industrial and Maritime Activity in the Thai Ocean

**Figure 2:**
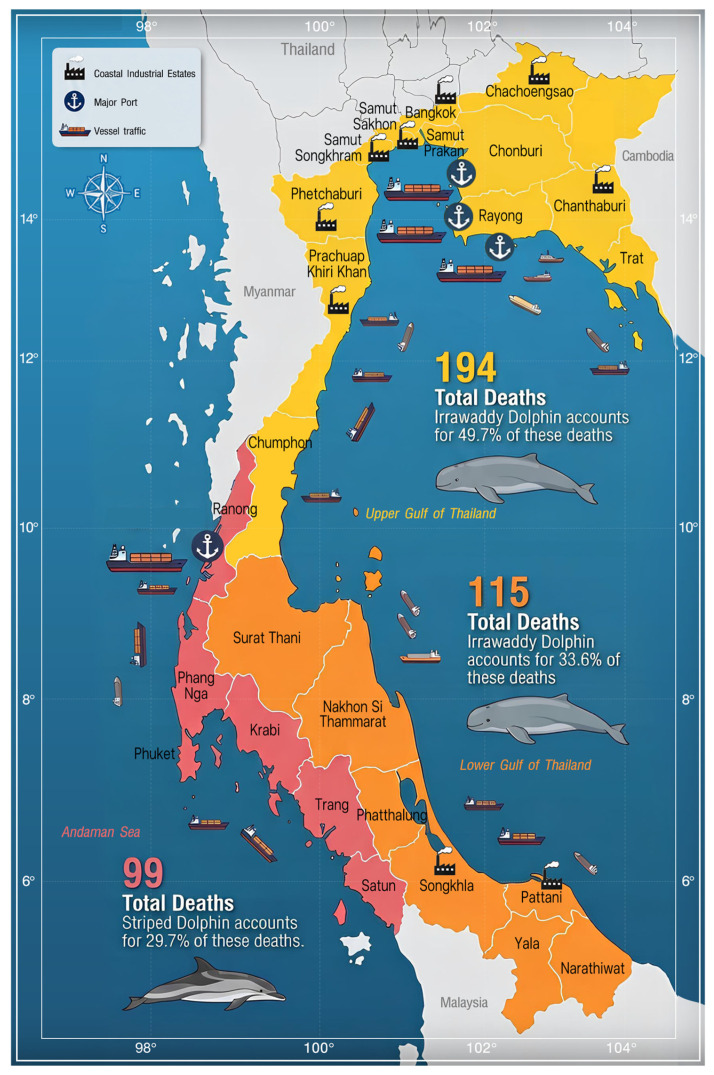
Mapping of coastal industrial estates, large ports (>5000 ships/year) and total deaths of dolphin in Thailand. Yellow provinces are the Upper Gulf of Thailand coast; oranges are the Lower Gulf of Thailand; and pinks are the Andaman Sea.

From the data of the Industrial Estate Authority of Thailand, there were 68 industrial estates across 16 provinces, of which 46 estates in six provinces are located in coastal areas. The majority of these coastal estates (*n* = 44, 95.65%) are concentrated in the Upper Gulf of Thailand (Figure 2), where the cetacean mortality rate was greater than other areas.

Vessel traffic data obtained from the Port Authority of Thailand and the Department of Marine showed a strong spatial concentration of maritime activity in the Upper Gulf of Thailand. A total of approximately 64,812 vessel movements and 338 million tonnes of cargo were recorded in this region, representing the majority of national maritime traffic. In contrast, vessel activity in the Lower Gulf of Thailand and the Andaman Sea was substantially lower, with approximately 2070 and 514 vessel movements, and 9 and 6 million tonnes of cargo, respectively. This spatial pattern corresponds with the regional distribution of cetacean mortality observed in the present study, in which the Upper Gulf exhibited the highest mortality rates. Based on this pronounced disparity in maritime activity, further quantitative analysis of vessel traffic was conducted specifically for the Upper Gulf of Thailand.

### 3.7. Regional Analysis of Factors Associated with Cetacean Mortality in the Andaman Sea

The initial national analysis revealed significant regional differences in cetacean mortality, likely reflecting variation in environmental conditions and anthropogenic pressures across the Andaman Sea, Upper Gulf of Thailand, and Lower Gulf of Thailand. Therefore, subregional analyses were conducted to identify region-specific drivers of mortality and to avoid masking important local patterns in pooled analyses.

In the Andaman Sea, univariable analysis identified several factors significantly associated (*p* < 0.05) with monthly cetacean mortality (Table S4). This included climatic seasonal variables such as land-based seasons (χ^2^ = 12.69, df = 2, *p* = 0.002), with mortality peaking during the rainy season (13.75 deaths/month; IRR = 4.23, *p* < 0.001). A similar pattern was observed for monsoon season (χ^2^ = 10.23, df = 2, *p* = 0.006), where mortality was significantly higher during the southwest monsoon (12.33 deaths/month; IRR = 2.85, *p* = 0.016) compared with the northeast monsoon. Notably, the southwest monsoon (May–October) largely overlaps with the rainy season (June–October), indicating a consistent seasonal pattern.

As seasonal classifications reflect the combined effects of multiple environmental variables, individual environmental factors were further analysed separately. Univariable analyses showed that rainfall and wind speed were significantly positively associated with cetacean mortality in the Andaman Sea (*p* ≤ 0.003), whereas sea surface temperature and salinity were not significant (*p* > 0.1) (Table S4). When analysed categorically, mortality increased progressively with higher rainfall levels (χ^2^ = 30.83, df = 3, *p* < 0.001), reaching 16.67 deaths/month at >200 mm (IRR = 5.56, *p* < 0.001). Similarly, rainfall as a continuous variable was significantly associated with mortality (IRR = 1.01 per mm increase, *p* = 0.003), with mortality increasing from 4.11 to 12.85 deaths/month across low to high rainfall levels. Wind speed also showed a significant positive association (χ^2^ = 8.94, df = 1, *p* = 0.003), corresponding to a 32% increase in mortality per unit increase (IRR = 1.32, *p* = 0.006), with mortality rising from 4.46 to 11.88 deaths/month across low to high wind conditions.

Among anthropogenic factors, tourism intensity was significantly associated with cetacean mortality in both categorical and continuous analyses (Table S4). Tourist season showed a significant effect (χ^2^ = 20.34, df = 2, *p* < 0.001), with the highest mortality observed during the low tourist season (13.80 deaths/month; IRR = 4.25, *p* < 0.001) compared with the high tourist season. Similarly, tourist numbers as a continuous variable were negatively associated with mortality (χ^2^ = 10.97, df = 1, *p* < 0.001), with mortality decreasing from 12.63 to 4.00 deaths/month as tourism increased (IRR = 0.99, *p* = 0.008). In contrast, fishery production was not significantly associated with mortality (*p* > 0.1).

A multivariable negative binomial regression model was statistically fit (χ^2^ = 13.01, df = 3, *p* = 0.005) incorporating significant continuous variables representing seasonal, environmental, and anthropogenic factors (Table 4). After adjustment, wind speed remained significantly positively associated with mortality (χ^2^ = 6.90, df = 1, *p* = 0.008), indicating a 22% increase (IRR = 1.22, *p* = 0.010) in mortality per 1 km/h increase in wind speed. Adjusted mortality increased from 4.78 to 9.75 deaths per month at the high wind speed (Table 4). In contrast, tourist numbers were significantly negatively associated with mortality (χ^2^ = 4.38, df = 1, *p* = 0.036), with adjusted mortality declining from 10.23 to 4.56 deaths per month at high tourist levels (IRR = 0.99, *p* = 0.039) (Table 4). However, rainfall volume was not significantly associated with mortality after adjustment (IRR = 1.00, *p* = 0.860), and predicted mortality remained relatively stable across rainfall levels. These findings indicate that wind speed and tourism intensity are independent predictors of cetacean mortality in the Andaman Sea, whereas the rainfall effect observed in univariable analysis likely reflects underlying seasonal variability.

### 3.8. Factors Associated with Cetacean Mortality in the Upper Gulf of Thailand

In addition to univariable analyses exploring associations between environmental, seasonal, and anthropogenic variables and monthly cetacean mortality—consistent with methodologies used in other regions—we specifically examined vessel traffic intensity in the Upper Gulf of Thailand. This inclusion was prompted by the region’s high density of maritime activity, centred around several major commercial hubs, most notably Laem Chabang Port, the largest and busiest port in Thailand.

**Figure 3:**
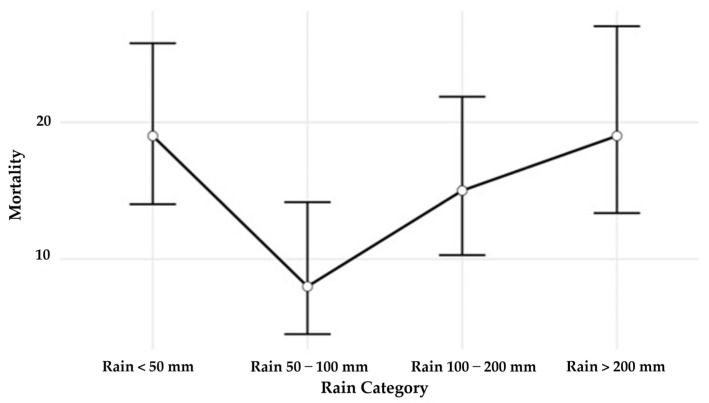
Predicted monthly cetacean mortality across rainfall categories in the Upper Gulf of Thailand based on a negative binomial regression model. Points represent estimated marginal means, and error bars indicate 95% confidence intervals.

Almost all variables were not significantly associated (*p* > 0.1) with cetacean mortality in the Upper Gulf of Thailand (Table S5). The exception was the rainfall category, which showed a significant association with monthly cetacean mortality (χ^2^ = 8.46, df = 3, *p* = 0.037). Mortality was significantly lower at 50–100 mm compared with <50 mm (IRR = 0.42, 95% CI: 0.22–0.80, *p* = 0.009), representing a 58% reduction (8 deaths/month). However, no clear dose–response pattern was observed across rainfall levels. Instead, the overall pattern suggested a non-linear (U-shaped) relationship, with higher mortality under both low (<50 mm) and high (>200 mm) rainfall conditions (Figure 3). This indicates that rainfall may act as a proxy for broader seasonal or oceanographic conditions rather than having a direct linear effect on mortality, with extreme conditions (drought and heavy rainfall) associated with increased risk.

### 3.9. Factors Associated with Cetacean Mortality in the Lower Gulf of Thailand

Univariable analysis for the Lower Gulf of Thailand indicated that several seasonal, environmental, and anthropogenic variables were significantly associated with cetacean mortality (*p* < 0.05; Table S6). Among the seasonal variables, land-based seasons showed a significant effect (χ^2^ = 8.46, df = 2, *p* = 0.015), with mortality peaking in the winter (13.50 deaths/month) and reaching its lowest point during the rainy season (6.50 deaths/month). This represents a 52% reduction in mortality during the rainy season (IRR = 0.48, *p* = 0.015). A consistent pattern emerged across monsoon periods (χ^2^ = 7.54, df = 2, *p* = 0.023); mortality was highest during the northeast monsoon (13.33 deaths/month) and lowest during the southwest monsoon (7.33 deaths/month), a 45% decrease (IRR = 0.55, *p* = 0.023). These findings are logically aligned, as the winter months (Nov–Feb) and the northeast monsoon (Nov–Jan) largely overlap in this region.

Regarding anthropogenic factors (Table S6), tourist season was significantly associated with mortality (χ^2^ = 9.11, df = 2, *p* = 0.011), while tourist numbers were not (*p* = 0.544). Compared to the high season (13.50 deaths/month), mortality decreased by 43–44% during the shoulder (7.67 deaths/month, IRR = 0.57, *p* = 0.036) and low seasons (7.60 deaths/month, IRR = 0.56, *p* = 0.028). In this region, which supports intensive dolphin-watching, the number of dolphin-watching boats was analysed and revealed that a significant negative association with mortality was observed (χ^2^ = 6.93, df = 1, *p* = 0.008). Mortality dropped from 12.30 deaths/month at low boat density to 6.97 deaths/month at high density (IRR = 0.99, *p* = 0.010).

**Figure 4:**
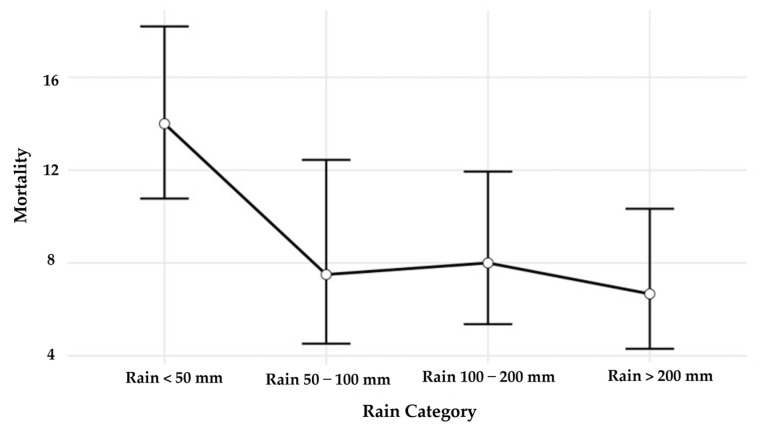
Predicted monthly cetacean mortality across rainfall categories in the Lower Gulf of Thailand based on a negative binomial regression model. Points represent estimated marginal means, and error bars indicate 95% confidence intervals.

Among environmental variables, sea surface temperature showed a significant negative association (χ^2^ = 9.85, df = 1, *p* = 0.017), with a 30% decrease in mortality per 1 °C increase (IRR = 0.70, *p* = 0.005) (Table S6). Conversely, wind speed was positively associated with mortality (χ^2^ = 6.38, df = 1, *p* = 0.033), showing a 28% increase per unit increase (IRR = 1.28, *p* = 0.012). Salinity and rainfall volume (as a continuous variable) showed no significant linear relationships with mortality (*p* > 0.1). However, categorical rainfall analysis revealed a significant association (χ^2^ = 11.91, df = 3, *p* = 0.007). Compared to low rainfall (<50 mm), mortality was significantly lower across all higher categories: 50–100 mm (IRR = 0.53), 100–200 mm (IRR = 0.57), and >200 mm (IRR = 0.47). This suggests a threshold effect, where mortality is markedly elevated only when rainfall is very low (<50 mm) (Figure 4).

The final multivariable model for the Lower Gulf of Thailand—comprising sea surface temperature, wind speed, and fishery production—was statistically significant (χ^2^ = 8.37, df = 3, *p* = 0.039). However, after adjustment, none of the individual predictors maintained a statistically significant association with cetacean mortality (*p* > 0.1, Table S7). This discrepancy suggests that mortality patterns likely reflect broader, integrated seasonal or oceanographic processes rather than the isolated influence of specific environmental or anthropogenic variables.

### 3.10. Regional Differences in Factors Associated with Cetacean Mortality

Marked regional differences in cetacean mortality (Tables S1–S7, Figure 1 and Figure 2) indicate that distinct environmental and anthropogenic drivers operate across the study areas. In the Andaman Sea, mortality was primarily driven by seasonal oceanographic factors—specifically wind speed—while tourism maintained a consistent negative association. Conversely, the Upper Gulf of Thailand yielded no consistent predictors, suggesting a complex system influenced by unmeasured factors, with the exception of a non-linear “U-shaped” association with rainfall. In the Lower Gulf, mortality was linked to seasonal, oceanographic, and anthropogenic variables in univariable analyses; however, no single factor remained independently significant in the multivariable model. This suggests that mortality in this region is driven by interacting processes rather than isolated predictors, as summarised in Table 5.

## 4. Discussion

### 4.1. Population Status and Regional Variation in Cetacean Mortality

The present study provides one of the first national-scale syntheses of cetacean population status and mortality trends in Thailand over approximately two decades. Notably, the estimated population of resident species increased between 2017 and 2023, followed by subsequent stabilisation. However, despite this apparent recovery, the conservation status of several key species remains of significant concern, particularly among coastal residents. Irrawaddy dolphins accounted for the majority of recorded mortalities, followed by finless porpoises and Indo-Pacific humpback dolphins. Importantly, cetacean mortality varied substantially among regions and was influenced by a combination of anthropogenic pressures and environmental or climatic factors.

Clear regional differences in dead species composition, with the striped dolphin most frequently recorded in the Andaman Sea and the Irrawaddy dolphin in the Gulf of Thailand, were found. This pattern is consistent with a recent national report in the Andaman Sea [1]. As oceanic cetaceans, the presence of the striped dolphin likely reflects the region’s steep bathymetry, which brings productive pelagic habitats closer to shore. This facilitates the co-occurrence of oceanic and coastal species and may increase their exposure to environmental and anthropogenic stressors. Additionally, high-density hotspots in the southeastern Indian Ocean—oceanographically connected to the Andaman Sea—may further contribute to the elevated stranding and death incidence in this region [29].

In particular, the dramatic decline of the Songkhla Lake (brackish) Irrawaddy dolphin population indicates an extremely high risk of local extinction, especially in enclosed aquatic environments. Similar declines in restricted habitats have been documented globally, including the Mekong River, Amazon River, and Ganges River [30,31,32]. This pattern highlights the vulnerability of small, isolated populations to genetic drift and inbreeding depression, both of which can compromise long-term viability and require targeted conservation strategies.

A notable finding was the discrepancy in regional mortality patterns between univariable and multivariable models. In univariable analyses, the Upper Gulf of Thailand exhibited the highest crude mortality, reflecting the observed distribution of carcass counts. However, after adjusting for environmental and anthropogenic covariates, the multivariable model revealed that the Andaman Sea carried the highest predicted baseline risk. This statistical divergence stems from a powerful regional confounding mechanism rather than a modelling artefact.

Unadjusted datasets are heavily biassed by regional detection probabilities and carcass wash-up dynamics. The high crude numbers in the Upper Gulf are largely driven by its shallow, semi-enclosed basin architecture, dense coastal human populations, and high nearshore observer presence, which collectively inflate carcass beaching rates and reporting efficiency. Conversely, the true baseline risk profile of the Andaman Sea is systematically masked in raw datasets by strong seasonal environmental conditions and human mobility interactions. Failing to statistically account for these covariates leads to an underestimation of baseline environmental hazards in open marine ecosystems, underscoring the necessity of relying on adjusted multivariable outputs to isolate true ecological drivers.

### 4.2. Drivers of High Cetacean Mortality in the Upper Gulf of Thailand

The present study identified the Upper Gulf of Thailand as the region with the highest cetacean mortality, a trend potentially driven by extreme weather and the area’s unique geography. As a shallow, semi-enclosed system, the Upper Gulf is heavily influenced by river discharge, intensive coastal development, and high vessel traffic intensity, all of which amplify environmental variability (Figure 2).

Crucially, although this region is the smallest and shallowest basin among the three studied regions, it yielded the highest absolute crude mortality counts across the two-decade record. This concentrates cetacean deaths relative to the basin’s size, proving that mortality is driven by dense local populations of resident coastal species and severe environmental anomalies within the basin. For marine conservation, this highlights the urgent need for targeted, high-resolution surveillance in smaller, high-density coastal areas rather than spreading resources uniformly across large geographic zones.

During periods of heavy rainfall, increased freshwater influx may alter coastal hydrography, enhance nutrient loading, and accelerate pollutant runoff. These factors can collectively compromise cetacean health, increase disease susceptibility, and disrupt prey availability [33,34,35]. Furthermore, enhanced coastal circulation during extreme weather events may facilitate the shoreward transport of carcasses. This suggests that the high mortality rates recorded may, in part, reflect inflated stranding data due to increased detection bias rather than a solely biological cause [36]. Conversely, extremely dry conditions may limit primary productivity by reducing nutrient input while simultaneously concentrating existing pollutants [37,38]. The resulting ecological stress and decreased prey availability are particularly impactful in shallow, semi-enclosed systems, especially for resident coastal species with limited movement flexibility [39,40].

This lack of significance contradicts the global importance of fisheries bycatch [24]. This discrepancy is largely due to our choice of data proxies. Specifically, our model used broad, macro-level economic metrics—such as total landed catch volume—rather than exact, fine-scale measurements like tracking fishing gear density, net soak times, or direct physical interaction frequencies. Consequently, these numbers reflect the region’s overall fish production capacity rather than the immediate, localised dangers of physical entanglement or vessel collisions.

Nevertheless, gillnets—particularly crab gillnets—are recognised as primary drivers of bycatch, accounting for most marine megafauna captures in the Gulf of Thailand [41,42], even though their independent effect was not statistically significant in our model. Similarly, across the northern Indian Ocean, nearshore gillnet fleets generate high, undocumented small cetacean bycatch loads that remain hidden within centralised macroeconomic statistics [43].

Notably, fishing gear encounters vary in severity; small cetaceans often break free or are released alive with non-lethal injuries or trailing gear [13,15]. These interactions cause sub-lethal stress, lacerations, or chronic entanglements that impair swimming and foraging without causing acute death [10,14]. Because our dataset relies strictly on secondary mortality records, these non-lethal events are omitted from the matrix. This limitation weakens the statistical association between broad fishery proxies and recorded deaths, as delayed or sub-lethal mortalities cannot be captured without fine-scale, longitudinal animal tracking [11,14].

The high mortality observed may also be influenced by population density, as the Upper Gulf supports relatively large resident populations of Irrawaddy dolphins and finless porpoises [44].

While the concentration of industrial estates and major ports poses a high risk of chemical pollution, existing data present a mixed picture. A local study in Trat Province—a high-mortality area—found that concentrations of copper, lead, and cadmium in the environment and cetacean tissues remained below regulatory limits [39]. In contrast, research in Chonburi—a province with even higher industrial density—reported elevated mercury levels in cetaceans, which may be a contributing factor to local mortality [45].

These mixed chemical pollution profiles across Thailand’s industrial coastlines mirror toxicological challenges in the Pearl River Delta. There, targeted risk assessments of stranded Sousa chinensis and finless porpoises demonstrated that heavy nearshore runoff results in the chronic ingestion of toxic polychlorinated compounds via contaminated prey [46].

Collectively, these findings suggest that cetacean mortality in this region is driven by the synergistic effects of multiple interacting stressors rather than a single dominant anthropogenic factor.

### 4.3. Influence of Environmental Variability

The present study demonstrates that environmental factors are the primary drivers of cetacean mortality at both national and regional levels; however, the specific influence of environmental variability differs significantly between regions.

In the Andaman Sea, wind speed emerged as a strong positive predictor of mortality, particularly during the southwest monsoon. Strong winds intensify wave energy and coastal circulation, which can alter prey distribution and significantly increase foraging costs. Furthermore, high-energy sea states can elevate the risk of navigation errors by disrupting echolocation in shallow waters, potentially leading to physical exhaustion and subsequent stranding and death [47,48]. Similar wind-driven mortality patterns have been documented in Southeast Australia [49] and Cape Cod, USA [50]. Additionally, wind-driven surface currents may enhance the shoreward transport of carcasses, thereby increasing detection rates in stranding and mortality records [51].

In the Lower Gulf of Thailand, mortality peaked during the northeast monsoon and winter months, where the synergistic effect of strong winds and low sea surface temperatures appears to drive cetacean mortality. Declining water temperatures can compromise the metabolic efficiency of coastal cetaceans, particularly individuals with insufficient blubber reserves [52,53]. Rapid thermal loss necessitates increased caloric intake to maintain homoeothermy; however, the accompanying turbulent conditions simultaneously increase foraging effort while dispersing prey clusters [54,55]. Furthermore, cold-water stress is a known driver of immunosuppression in tropical marine mammals, potentially increasing susceptibility to opportunistic pathogens or exacerbating underlying health conditions [56]. Juvenile cetaceans are particularly susceptible to stranding and mortality as they often lack the physical strength and thermal insulation required to cope with high-energy sea states and low water temperatures [11,52,53].

### 4.4. Anthropogenic Pressures: Tourism and Fisheries

Anthropogenic activities exhibited complex, region-specific relationships with cetacean mortality. Nationally and within the Andaman Sea, tourism intensity was negatively associated with mortality. This pattern likely reflects seasonal confounding; peak tourism in Thailand coincides with calmer sea states, which reduce natural environmental stressors [57]. Furthermore, marine ecotourism increases environmental surveillance. Active monitoring by operators, fishermen, and authorities enables early live-stranding detection—improving rescue outcomes—while deterring illegal fishing [58].

Interestingly, fishery production had no statistically significant association with cetacean mortality. Although high production occurs during calm sea states and can signify increased fishing activity, this lack of significance aligns with previous Andaman Sea research showing that natural causes dominate cetacean mortality, while fisheries account for only ~5% of documented cases [1,59].

Regarding specialised tourism, dolphin-watching can alter cetacean behaviour by reducing resting time and increasing vocalisation or avoidance movements [24,25]. However, because this activity occurs during the calm season when mortality is naturally lower, a higher number of dolphin-watching boats was negatively associated with mortality. While favourable weather currently overrides tourism-related stressors, potential long-term cumulative impacts on population fitness warrant further high-resolution physiological study.

Biologically and logistically, these favourable environmental conditions suppress raw stranding counts through two climate-mediated mechanisms. First, reduced hydrodynamic energy during peak tourism limits the calm nearshore transport of drifting offshore carcasses to accessible coastlines, causing them to sink or decompose undetected [36,51]. Second, an intense observer detection bias is created by the massive influx of seasonal tourists, which ensures that the few carcasses reaching the shore are reported immediately, artificially inflating local detection probabilities [11,51].

During the rough southwest monsoon, this dynamic completely inverts as strong monsoonal winds accelerate the shoreward transport of carcasses onto complex, rocky coastlines where public presence and reporting drop to near zero [18,51]. By incorporating tourism volume as a localised covariate, our multivariable model successfully filters out these severe observer and transport biases to isolate the true baseline risk. This demonstrates that cetacean populations in the Andaman Sea face a higher continuous baseline environmental hazard than raw stranding data suggest [11,15].

### 4.5. Comparative Regional Frameworks and Technical Innovations

To maximise the efficacy of national marine governance, Thailand’s cetacean reporting architecture must be contextualised within the broader marine networks of the Indian and Pacific Oceans, which connect directly to the Andaman Sea and the Gulf of Thailand. The multi-decadal upward trajectory in crude cetacean mortality documented in Thai waters closely aligns with wider trends observed across the Northwest Pacific. For instance, long-term baseline registries in mainland China have revealed a steady temporal increase in stranding frequencies, heavily driven by intensifying nearshore human activities and expanding economic development.

Similarly to the Upper Gulf of Thailand, highly urbanised and industrial estuaries in China’s Pearl River Delta face severe ecological pressures, including small cetacean displacement, toxic chemical bioaccumulation, heavy commercial vessel noise, and construction runoff [46,60]. This chemical pollution is positively associated with cetacean mortality and an elevated risk of parasitic infections [61,62], likely due to contaminant-induced immunosuppression. These patterns indicate that the stress generated by vessel traffic noise and chemical pollution compounds baseline mortality rates, operating alongside dominant climatic and environmental drivers [60].

Concurrently, the enforcement of a ten-year fishing ban in China’s Yangtze River has successfully facilitated the recovery and population increase in the local, critically endangered Yangtze finless porpoise [63]. A comparable, targeted closure strategy may represent a highly viable intervention framework to rescue distressed cetacean populations in restricted Thai waters—most notably within Songkhla Lake, where the isolated resident population has collapsed to just 14 individuals.

While macro-level variables like regional port cargo volumes did not yield a statistically significant independent effect in our model, this alignment with previous research in the Andaman Sea indicates that cetacean mortality from ship-related collision trauma remains rarer than natural causes in this region [1]. However, localised maritime activities cannot be dismissed as stressors, as they generate persistent noise pollution that can lead to navigational disorientation and internal acoustic injuries [64]. These anthropogenic pressures may ultimately compound baseline physiological stress, thereby increasing a cetacean’s vulnerability to mortality from climate change and natural infections.

This environment-driven mortality hypothesis is strongly reinforced by satellite-derived oceanographic indicators from the Indian southwest coast, where long-term analyses demonstrate a significant positive correlation between stranding events and chlorophyll-a concentrations, alongside a significant negative correlation with SST [65]. This confirms that monsoon-driven upwelling forces nutrient-rich deep water to the surface, sparking dense phytoplankton and pelagic fish aggregations that attract large cetaceans close to the shoreline, thereby escalating their exposure to localised coastal hazards and vessel traffic [65].

Implementing advanced postmortem imaging would effectively circumvent these diagnostic limitations, especially since vast baseline data gaps persist across Indian Ocean coastal zones due to a structural historical reliance on opportunistic beach recordings rather than rigorous visual, acoustic, or digital tracking surveys [66]. A critical advancement that should be adapted is the systematic integration of postmortem advanced imaging techniques, commonly referred to as “virtopsy” approaches, which have already been successfully implemented in cetacean stranding programmes [67]. Relying primarily on classic, open-air descriptive field autopsies often leaves deep internal injuries unrecorded or severely compromised, particularly when managing carcasses with advanced postmortem decomposition [67]. Adopting non-invasive computed tomography or magnetic resonance imaging protocols allows investigators to cleanly isolate sublethal lesions including acoustic trauma, internal barotrauma, neurological pathologies, and hidden bone fractures that may increase risk of mortality [67,68].

### 4.6. Study Limitations

While this study provides the first integrated, national-scale synthesis of long-term cetacean mortality in Thai waters, several inherent methodological limitations must be acknowledged. First, the analysis relied primarily on secondary data sources compiled from citizen-based networks, media monitoring, and official government incident logs. Consequently, these historical records are heavily influenced by temporal reporting effort, physical coastline accessibility, and fluctuating public conservation awareness. Such factors inevitably introduce data-collection inequalities across different regions, potentially leading to the under- or overestimation of true baseline mortality rates—a phenomenon widely recognised as detection or observer bias.

Second, the consolidated dataset aggregates an array of heterogeneous mortality typologies, including coastal live strandings, physical bycatch entanglements, and drifting offshore carcasses. These distinct categories carry highly disparate detection probabilities and are driven by fundamentally different underlying aetiologies. Furthermore, the lack of systematic, standardised necropsy data across the two-decade timeline constrained our ability to definitively establish proximate and ultimate causes of death. This diagnostic limitation is particularly challenging when attempting to rigorously distinguish natural systemic pathologies from cryptic, anthropogenic trauma.

Third, the environmental and oceanographic datasets applied possessed relatively coarse spatial and temporal resolutions. This lack of microgeographic granularity may limit the negative binomial regression models’ capacity to detect fine-scale, transient, or highly localised relationships between rapid environmental shifts and immediate cetacean mortality responses.

Finally, the reliance on indirect socioeconomic proxies—specifically aggregated fishery production tonnage and regional port cargo volumes—presents a structural limitation. These macro-level indicators reflect broad regional economic outputs rather than the true spatial intensity, localised net deployment distributions, or direct, real-time physical interactions between maritime activities and cetaceans. This lack of high-resolution operational effort data likely introduced a masking effect within our multivariable equations, potentially obscuring more direct, fine-scale associations that may operate at a localised or habitat-specific level.

### 4.7. Implications for Conservation and Monitoring

Despite these limitations, this study provides important insights into the spatiotemporal dynamics of cetacean mortality in Thai waters and highlights key conservation priorities. The Upper Gulf of Thailand should be considered a high-risk region requiring enhanced monitoring and targeted mitigation; however, no dominant driver was identified, suggesting the influence of complex, interacting factors that warrant further investigation. Given the region-specific nature of mortality patterns, management strategies should be tailored accordingly. In the Andaman Sea and the Lower Gulf of Thailand, where mortality appears strongly influenced by climatic variability, intensified monitoring and stranding response efforts are particularly needed during peak-risk periods.

### 4.8. Future Research Directions

Future research should focus on clarifying currently uninvestigated mechanisms underlying cetacean mortality and strengthening monitoring systems in Thai waters. Expanding health surveillance through routine pathogen screening in carcasses and seawater is essential to assess the role of infectious diseases in mortality [22]. In addition, toxicological analyses and assessments of plastic debris in the environment [27], prey species, and carcasses—particularly in areas with high industrial activity or petroleum infrastructure—are needed to evaluate the effects of pollution on cetacean health and mortality. Improved assessment of anthropogenic pressures using spatially explicit data (e.g., fishing effort, gear distribution, and automatic identification system (AIS) vessel tracking) is also needed to better quantify human–cetacean interactions.

Integrating oceanographic processes with stranding and death data, including carcass drift modelling, would help distinguish true mortality hotspots from detection bias. In addition, long-term ecological monitoring such as photo-identification, passive acoustic monitoring, and habitat-use studies should be linked with environmental and prey data to better understand population dynamics. Finally, strengthening national monitoring systems, including citizen-based reporting networks, would improve detection, data quality, and early response to mortality events.

## 5. Conclusions

This study provides the first national-scale synthesis of cetacean population status and mortality in Thailand over two decades, documenting 29 species and highlighting the high marine mammal diversity in Thai waters.

Mortality patterns varied markedly among regions, with the Upper Gulf of Thailand exhibiting the highest mortality, likely driven by multiple interacting factors. In contrast, environmental variability, particularly wind speed, played a more prominent role in shaping mortality patterns in the Andaman Sea and the Lower Gulf. Anthropogenic factors, particularly tourism, showed a significant negative association with cetacean mortality, suggesting that higher tourism activity may not be a primary driver of mortality. Overall, these findings emphasise the importance of region-specific approaches and improved monitoring systems to better understand and mitigate cetacean mortality in Thai waters.

## Figures and Tables

**Table 1:** Cetaceans recorded in Thailand (29 species) and their death records (*n* = 370).

Ecological Group	Common Name	Scientific Name	Family	Death %(*n*)	CITES	IUCN Status	
						Global	Thailand
Coastal residents (total death = 298, 80.54%)	Indo-Pacific bottlenose dolphin	*Tursiops aduncus*	Delphinidae	8.11 (30)	II	NT	EN
	Finless porpoise	*Neophocaena phocaenoides*	Phocoenidae	18.38 (68)	I	VU	EN
	Indo-Pacific humpback dolphin	*Sousa chinensis*	Delphinidae	8.92 (33)	I	VU	EN
	Irrawaddy dolphin	*Orcaella brevirostris*	Delphinidae	37.30 (137)	I	EN	EN
	Bryde’s whale	*Balaenoptera edeni*	Balaenopteridae	6.76 (25)	I	LC	EN
	Omura’s whale	*Balaenoptera omurai*	Balaenopteridae	1.35 (5)	I	DD	NE
Oceanic baleen whales (total death = 1, 0.27%)	Blue whale	*Balaenoptera musculus*	Balaenopteridae	0.00 (0)	I	EN	NE
	Fin whale	*Balaenoptera physalus*	Balaenopteridae	0.00 (0)	I	VU	NE
	Humpback whale	*Megaptera novaeangliae*	Balaenopteridae	0.00 (0)	I	LC	NE
	Common minke whale	*Balaenoptera acutorostrata*	Balaenopteridae	0.27 (1)	I	LC	NE
Oceanic delphinids (total death = 59, 15.94%)	False killer whale	*Pseudorca crassidens*	Delphinidae	1.35 (5)	II	NT	NE
	Risso’s dolphin	*Grampus griseus*	Delphinidae	0.27 (1)	II	LC	NE
	Short-finned pilot whale	*Globicephala macrorhynchus*	Delphinidae	0.27 (1)	II	LC	NE
	Fraser’s dolphin	*Lagenodelphis hosei*	Delphinidae	1.08 (4)	II	LC	NE
	Long-beaked common dolphin	*Delphinus capensis*	Delphinidae	0.54 (2)	II	LC	NE
	Pygmy killer whale	*Feresa attenuata*	Delphinidae	0.27 (1)	II	NT	NE
	Killer whale	*Orcinus orca*	Delphinidae	0.27 (1)	II	DD	NE
	Melon-headed whale	*Peponocephala electra*	Delphinidae	0.54 (2)	II	LC	NE
	Pantropical spotted dolphin	*Stenella attenuata*	Delphinidae	0.54 (2)	II	LC	NE
	Rough-toothed dolphin	*Steno bredanensis*	Delphinidae	0.81 (3)	II	LC	NE
	Striped dolphin	*Stenella coeruleoalba*	Delphinidae	6.76 (25)	II	LC	NE
	Spinner dolphin	*Stenella longirostris*	Delphinidae	3.24 (12)	II	LC	NE
Oceanic odontocetes (total death = 12, 3.24%)	Dwarf sperm whale	*Kogia sima*	Kogiidae	1.08 (4)	II	LC	NE
	Pygmy sperm whale	*Kogia breviceps*	Kogiidae	0.00 (0)	II	LC	NE
	Sperm whale	*Physeter macrocephalus*	Physeteridae	1.35 (5)	I	VU	NE
	Blainville’s beaked whale	*Mesoplodon densirostris*	Ziphiidae	0.27 (1)	II	LC	NE
	Cuvier’s beaked whale	*Ziphius cavirostris*	Ziphiidae	0.27 (1)	II	LC	NE
	Ginkgo-toothed beaked whale	*Mesoplodon ginkgodens*	Ziphiidae	0.00 (0)	II	DD	NE
	Longman’s beaked whale	*Indopacetus pacificus*	Ziphiidae	0.27 (1)	II	DD	NE

CITES: Appendix I = International commercial trade prohibited; Appendix II = Trade regulated under permit system. IUCN Abbreviations: EN = Endangered; VU = Vulnerable; NT = Near Threatened; LC = Least Concern; DD = Data Deficient (IUCN Red List categories). Unknown species was report as 9.31% (*n* = 38).

**Table 2:** Regional differences in monthly cetacean mortality.

Predictor (Region)	IRR (95% CI)	EMM (Deaths/Month, 95% CI)	*p*-Value
Andaman (reference)	1.00	8.25 (5.99–11.37)	–
Upper Gulf of Thailand	1.96 (1.27–3.02)	16.17 (12.11–21.59)	0.002
Lower Gulf of Thailand	1.16 (0.74–1.82)	9.58 (7.02–13.09)	0.512

*p* < 0.05 is considered a statistically significant difference. IRR = Incidence Rate Ratios. CI = Confidential Interval. EMM = Estimated Marginal Means of mortality.

**Table 3:** Final multivariable negative binomial regression model of factors associated with national cetacean mortality.

Predictor	Category/Comparison	IRR (95% CI)	*p*-Value	Adjusted Mortality (95% CI)
Region χ^2^ = 9.74, df = 2, *p* = 0.008	Andaman Sea (reference)	1.00	—	56.08 (15.00–209.62)
	Upper Gulf of Thailand	0.07 (0.01–0.67)	0.020	4.13 (1.64–10.35)
	Lower Gulf of Thailand	0.09 (0.01–0.53)	0.008	4.86 (2.81–8.41)
Tourism number χ^2^ = 9.54, df = 1, *p* = 0.002	Per 1000 tourists increase	0.99 (0.99–1.00)	0.003	—
	Low (−1 SD)	—	—	47.33 (17.38–128.91)
	Mean	—	—	10.40 (8.88–12.17)
	High (+1 SD)	—	—	2.28 (0.80–6.53)
Rainfall volume χ^2^ = 0.09, df = 1, *p* = 0.757	Per 1 mm increase	1.00 (0.99–1.00)	0.761	—
	Low (−1 SD)	—	—	10.10 (8.07–12.70)
	Mean	—	—	10.40 (8.88–12.20)
	High (+1 SD)	—	—	10.70 (8.48–13.40)

IRR = Incidence Rate Ratios. SD = Standard Deviation. CI = Confidential Interval.

**Table 4:** Multivariable negative binomial regression of monthly cetacean mortality in the Andaman Sea.

Predictor/Statistic Model	Category/Comparison	IRR (95% CI)	Adjusted Mortality (95% CI)	*p*-Value
Rainfall volume	Per 1 mm increase	1.00 (0.99–1.00)	-	0.860
χ^2^ = 0.03, df = 1, *p* = 0.860	Low (−1 SD)	-	6.59 (4.08–10.65)	-
	Mean	-	6.83 (5.32–8.77)	-
	High (+1 SD)	-	7.08 (4.50–11.14)	-
Wind speed	Per 1 km/h increase	1.22 (1.05–1.43)	-	0.010
χ^2^ = 6.90, df = 1, *p* = 0.008	Low (−1 SD)	-	4.78 (3.21–7.12)	-
	Mean	-	6.83 (5.32–8.77)	-
	High (+1 SD)	-	9.75 (6.96–13.66)	-
Tourist numbers	Per 1000 tourists increase	0.99 (0.99–1.00)	-	0.039
χ^2^ = 4.38, df = 1, *p* = 0.036	Low (−1 SD)	-	10.23 (6.71–15.59)	-
	Mean	-	6.83 (5.32–8.77)	-
	High (+1 SD)	-	4.56 (2.79–7.46)	-

Model statistics: χ^2^ = 13.01, df = 3, *p* = 0.005. IRR = Incidence Rate Ratios. SD = Standard Deviation. CI = Confidential Interval.

**Table 5:** Multivariable negative binomial regression analysis of factors associated with monthly cetacean mortality in the Lower Gulf of Thailand.

Region	Key Significant Factors (Univariable)	Significant Factors (Multivariable)	Direction of Effect	Overall Interpretation
Andaman Sea	Season (rainy, SW monsoon), rainfall, wind speed, tourism	Wind speed (+), tourism (−)	High wind increased mortality; Low tourism decreased mortality	Mortality mainly driven by environmental/seasonal dynamics; tourism shows protective association
Upper Gulf of Thailand	Rainfall (non-linear; both low and high extremes)	None	Extreme weather (too little vs. too much rainfall)	No consistent predictors identified. Mortality may be influenced by complex or unmeasured factors, with only a non-linear association with rainfall observed.
Lower Gulf of Thailand	Season, SST (−), wind speed (+), fishery production (−), rainfall (threshold)	None	Mixed effects	Mortality influenced by combined environmental and anthropogenic factors; no independent predictors after adjustment

SW = southwest, SST = sea surface temperature.

## Data Availability

The original data presented in the study are openly available in Mendeley at https://data.mendeley.com/datasets/ct95kksrd2/1, accessed on 1 June 2026.

## Supplementary Materials

The following supporting information can be downloaded at: https://www.mdpi.com/article/10.3390/ani16111733/s1, Table S1. Estimation of resident cetaceans in Thai water. Table S2. Total number of cetacean mortality comparison between regions. Table S3. National associations between environmental and anthropogenic variables and cetacean mortality (univariable negative binomial regression models). Table S4. Univariable analysis of factors associated with monthly cetacean mortality in the Andaman Sea. Table S5. Unviable analysis of factors associated with cetacean mortality in the Upper Gulf of Thailand. Table S6. Univariable analysis of factors associated with monthly cetacean mortality in the Lower Gulf of Thailand. Table S7. Multivariable negative binomial regression analysis of factors associated with monthly cetacean mortality in the Lower Gulf of Thailand.
