# Supplementary material for: Two Decades of Cetacean Population Status and Mortality in Thailand: Spatiotemporal Trends, Environmental Drivers, and Anthropogenic Stressors
Source: Animals (Basel). 2026 Jun 4;16(11):1733. doi: 10.3390/ani16111733 (PMC13255866; doi:10.3390/ani16111733)
Supplement: Supplementary file 1 [file animals-16-01733-s001.zip › animals-4304987-supplementary.pdf]

Table S1. Estimation of resident cetaceans in Thai water

Year	Indo-Pacific bottlenose dolphin	Finless porpoise	Indo-Pacific humpback dolphin	Irrawaddy dolphin	Bryde's whale	Omura's whale	Total
2017	90	433	433	534	62	2	1,554
2018	190	670	588	635	60	3	2,146
2019	190	695	566	660	70	2	2,183
2020	357	675	591	860	59	3	2,545
2021	513	557	513	624	66	2	2,272
2022	249	705	617	679	60	2	2,312
2023	480	887	529	1,030	71	5	3,002
2024*	450	850	520	950	70	10	2,850
2025*	430	820	500	900	70	5	2,725

*Estimates for 2024 and 2025 represent projected population trajectories based on the 2023 peak and subsequent mortality rates.

Table S2. Total number of cetacean mortality comparison between regions.

Common name	Andaman Sea		Upper Gulf of Thailand		Lower Gulf of Thailand		Total	
	Number	%	Number	%	Number	%	Number	%
Irrawaddy dolphin	12	14.29	89	49.72	36	33.64	137	37.03
Indo-Pacific humpback dolphin	4	4.76	4	2.23	25	23.36	33	8.92
Finless porpoise	3	3.57	45	25.14	20	18.69	68	18.38
Indo-Pacific bottlenose dolphin	6	7.14	15	8.38	9	8.41	30	8.11
Bryde's whale	2	2.38	16	8.94	7	6.54	25	6.76
Spinner dolphin	9	10.71	1	0.56	2	1.87	12	3.24
Omura's whale	1	1.19	2	1.12	2	1.87	5	1.35
Pantropical spotted dolphin	0	0.00	0	0.00	2	1.87	2	0.54
Sperm whale	4	4.76	0	0.00	1	0.93	5	1.35
Fraser's dolphin	3	3.57	0	0.00	1	0.93	4	1.08
False killer whale	0	0.00	4	2.23	1	0.93	5	1.35
Blainville's beaked whale	0	0.00	0	0.00	1	0.93	1	0.27
Striped dolphin	25	29.76	0	0.00	0	0.00	25	6.76
Dwarf sperm whale	4	4.76	0	0.00	0	0.00	4	1.08
Rough-toothed dolphin	3	3.57	0	0.00	0	0.00	3	0.81
Long-beaked common dolphin	2	2.38	0	0.00	0	0.00	2	0.54
Melon-headed whale	2	2.38	0	0.00	0	0.00	2	0.54
Risso's dolphin	1	1.19	0	0.00	0	0.00	1	0.27
Short-finned pilot whale	1	1.19	0	0.00	0	0.00	1	0.27
Pygmy killer whale	1	1.19	0	0.00	0	0.00	1	0.27
Cuvier's beaked whale	1	1.19	0	0.00	0	0.00	1	0.27
Common minke whale	0	0.00	1	0.56	0	0.00	1	0.27
Killer whale	0	0.00	1	0.56	0	0.00	1	0.27
Longman's beaked whale	0	0.00	1	0.56	0	0.00	1	0.27

**Total**	**84**	**100.00**	**179**	**100.00**	**107**	**100.00**	**370**	**100.00**

Table S3. National associations between environmental and anthropogenic variables and cetacean mortality (univariable negative binomial regression models).

Predictor	Variable	X^2	df	p-value	Interpretation
Region	Categorical	10.89	2	0.004	Significant association
Monsoon season	Categorical	0.85	2	0.653	No significant association
Land season	Categorical	0.30	2	0.856	No significant association
Tourist season	Categorical	3.19	2	0.202	No significant association
Rainfall	Continuous	3.70	1	0.054	Marginal association
Sea temperature	Continuous	2.47	1	0.116	No significant association
Wind speed	Continuous	0.06	1	0.792	No significant association
Salinity	Continuous	1.64	1	0.199	No significant association
Tourist number	Continuous	9.99	1	0.001	Significant association
Fishery production	Continuous	1.95	1	0.161	No significant association

$p < 0.05$ is considered statistically significant, $P = 0.05$-0.10 is marginal trend. $P > 0.10$ not statistically significant.

Table S4. Univariable analysis of factors associated with monthly cetacean mortality in the Andaman Sea

Predictor/Statistic model	Category / Comparison	IRR (95% CI)	EMM/PMM (95% CI)	p-value
Land-based season	Winter (reference)	1.00	3.25 (1.66–6.35)	–
$\chi^2 = 12.69$, df = 2, $P = 0.002$	Summer	2.38 (1.02–5.60)	7.75 (4.57–13.13)	0.046
	Rainy	4.23 (1.86–9.61)	13.75 (8.57–22.07)	< 0.001
Monsoon season	Northeast monsoon (reference)	1.00	4.33 (2.07–9.05)	–
$\chi^2 = 10.23$, df = 2, $P = 0.006$	Dry / inter-monsoon	0.92 (0.32-2.64)	4.00 (1.88–8.50)	0.881
	Southwest monsoon	2.85 (1.22–6.64)	12.33 (8.11–18.75)	0.016
Tourist season	High tourist season (reference)	1.00	3.25 (1.76–6.01)	–
$\chi^2 = 20.34$, df = 2, $P < 0.001$	Shoulder season	1.74 (0.74-4.05)	5.66 (3.17-10.11)	0.197

Predictor/Statistic model	Category / Comparison	IRR (95% CI)	EMM/PMM (95% CI)	p-value
	Low tourist season	4.25 (2.09–8.61)	13.80 (9.74–19.56)	< 0.001
Rainfall (categorical)	<50 mm (reference)	1.00	3.00 (1.66-5.41)	–
$\chi^2 = 30.83$, df = 3, P < 0.001	50–100 mm	2.16 (0.93-5.00)	6.50 (3.59-11.75)	0.070
	100–200 mm	2.66 (1.27-5.58)	8.00 (5.13-12.47)	0.009
	>200 mm vs <50 mm	5.56 (2.82–10.96)	16.67 (11.89-23.36)	< 0.001
Rainfall (continuous)	Per 1 mm increase	1.01 (1.00–1.01)	–	0.003
$\chi^2 = 7.85$, df = 1, P = 0.005	Low (-1 SD)	–	4.11 (2.31-7.30)	–
	Mean	–	7.27 (5.07-10.42)	–
	High (+1 SD)	–	12.85 (8.02-20.57)	–
Wind speed	Per 1 km/h increase	1.32 (1.08–1.61)	–	0.006
$\chi^2 = 8.94$, df = 1, P = 0.003	Low (-1 SD)	–	4.46 (2.63-7.58)	–
	Mean	–	7.28 (5.21-10.17)	–
	High (+1 SD)	–	11.88 (7.67-18.39)	–
Tourist numbers	Per 1000 tourists increase	0.99 (0.99-1.00)	–	0.008
$\chi^2 = 10.97$, df = 1, P < 0.001	Low (-1 SD)	–	12.63 (8.26-19.31)	–
	Mean	–	7.11 (5.13-9.86)	–
	High (+1 SD)	–	4.00 (2.30-6.97)	–
Sea surface temperature	Per 1 °C increase	1.16 (0.52-2.62)	–	0.712
$\chi^2 = 0.13$, df = 1, P = 0.71	Low (-1 SD)	–	7.51 (3.91-14.41)	–
	Mean	–	8.22 (5.25-12.85)	–
	High (+1 SD)	–	8.99 (4.73-17.07)	–
Salinity	Per 1 ppt increase	0.91 (0.48-1.74)	–	0.771
$\chi^2 = 0.08$, df = 1, P = 0.77	Low (-1 SD)	–	8.76 (4.61-16.65)	–
	Mean	–	8.23 (5.25-12.88)	–
	High (+1 SD)	–	7.73 (4.04-14.79)	–
Fishery production	Per 1000 tons increase	0.99 (0.98-1.00)	–	0.149
$\chi^2 = 2.32$, df = 1, P = 0.13	Low (-1 SD)	–	10.89 (6.13-19.36)	–

Predictor/Statistic model	Category / Comparison	IRR (95% CI)	EMM/PMM (95% CI)	p-value
	Mean	–	7.83 (5.19-11.82)	–
	High (+1 SD)	–	5.64 (3.02-10.51)	–

IRR = Incidence Rate Ratios. SD = Standard Deviation. CI = Confidential Interval. PMM = Predicted Mean Mortality. EMM = Estimated Marginal Means of mortality.

Table S5. Unviable analysis of factors associated with cetacean mortality in the Upper Gulf of Thailand

**Variables**	**Statistics**
Land-based season	$\chi^2 = 0.03$, df = 2, p = 0.988
Monsoon season	$\chi^2 = 0.24$, df = 2, p = 0.886
Tourist season	$\chi^2 = 2.05$, df = 2, p = 0.358
Tourist numbers	$\chi^2 = 0.001$, df = 1, p = 0.973
Rainfall volume	$\chi^2 = 0.52$, df = 1, p = 0.472
Sea surface temperature	$\chi^2 = 0.64$, df = 1, p = 0.425
Salinity	$\chi^2 = 0.25$, df = 1, p = 0.612
Wind speed	$\chi^2 = 1.66$, df = 1, p = 0.197
Fishery production	$\chi^2 = 0.40$, df = 1, p = 0.522
Vessel traffic intensity	$\chi^2 = 1.23$, df = 1, p = 0.267
Rainfall category	$\chi^2 = 8.46$, df = 3, p = 0.037

Table S6. Univariable analysis of factors associated with monthly cetacean mortality in the Lower Gulf of Thailand

**Predictor/Statistic model**	**Category / Comparison**	**IRR (95% CI)**	**EMM/PMM (95% CI)**	**p-value**
**Land-based season**	Winter	1.00 (reference)	13.50 (10.34–17.63)	–
**$\chi^2 = 8.46$, df = 2, p = 0.015**	Summer vs Winter	0.65 (0.39–1.07)	8.75 (6.28–12.19)	0.092
	Rainy vs Winter	0.48 (0.27–0.86)	6.50 (4.43–9.55)	0.015
**Monsoon season**	Northeast monsoon (NE)	1.00 (reference)	13.33 (9.86–18.03)	–
**$\chi^2 = 7.54$, df = 2, p = 0.023**	Dry/inter-monsoon vs NE	0.71 (0.40–1.27)	9.50 (6.20–14.54)	0.241
	Southwest monsoon vs NE	0.55 (0.32–0.95)	7.33 (4.98–10.78)	0.023
**Tourist season**	High season	1.00 (reference)	13.50 (10.01–18.21)	–
**$\chi^2 = 9.11$, df = 2, p = 0.011**	Shoulder vs High	0.57 (0.34–0.96)	7.67 (5.18–11.35)	0.036
	Low vs High	0.56 (0.33–0.94)	7.60 (5.10–11.32)	0.028
**Sea surface temperature**	per 1 °C increase	0.70 (0.54–0.90)	-	0.005
**$\chi^2 = 9.85$, df = 1, p = 0.017**	Low (-1 SD)	-	12.42 (9.36–16.48)	-
	Mean	-	9.18 (7.59 –11.11)	-
	High (+1 SD)	-	6.79 (4.89–9.42)	-
**Wind speed**	Per 1 km/h increase	1.28 (1.06–1.55)	-	0.012
**$\chi^2 = 6.38$, df = 1, p = 0.033**	Low (-1 SD)	-	7.00 (5.01–9.79)	-
	Mean	-	9.58 (7.39 –11.76)	-
	High (+1 SD)	-	12.17 (8.79–16.85)	-
**Fishery production**	Per 1000 tons increase	0.84 (0.75–0.94)	-	0.004
**$\chi^2 = 8.25$, df = 1, p = 0.004**	Low (-1 SD)	-	12.14 (9.67–15.42)	-

	Mean	-	9.25 (7.66 –11.17)	-
	High (+1 SD)	-	7.05 (5.24–9.47)	-
**Rainfall (continuous)**	per 1 mm increase	1.00 (0.99–1.00)	-	0.412
**χ^2 = 0.08, df = 1, p = 0.767**	Low (-1 SD)	-	9.93 (7.01–14.05)	-
	Mean	-	9.57 (7.52–12.19)	-
	High (+1 SD)	-	9.23 (6.47–13.15)	-
**Rainfall (categorical)**	<50 mm	1.00 (reference)	14.00 (10.77-18.19)	
**χ^2 = 11.91, df = 1, p = 0.007**	50–100 mm	0.53 (0.30-0.94)	7.50 (4.52-12.44)	0.031
	100–200 mm	0.57 (0.35-0.92)	8.00 (5.36-11.93)	0.021
	>200 mm	0.47 (0.28-0.79)	6.66 (4.30-10.33)	0.004
**Salinity**	per 1 ppt increase	0.97 (0.82–1.14)	-	0.771
**χ^2 = 1.37, df = 1, p = 0.242**	Low (-1 SD)	-	11.52 (7.69–15.34)	-
	Mean	-	10.09 (7.45–12.72)	-
	High (+1 SD)	-	8.66 (4.84–12.48)	-
**Tourist number**	Per 1000 tourists increase	0.99 (0.96–1.02)	-	0.294
**χ^2 = 0.36, df = 1, p = 0.544**	Low (-1 SD)	-	10.30 (7.35–14.42)	-
	Mean	-	9.55 (7.53–12.12)	-
	High (+1 SD)	-	8.86 (6.23–12.59)	-
**Dolphin tour (boat number)**	Per 1 boat increase	0.99 (0.99-1.00)		0.010
**χ^2 = 6.93, df = 1, p = 0.008**	Low (-1 SD)	–	12.29 (9.52-15.87)	–
	Mean	–	9.25 (7.59-11.27)	–
	High (+1 SD)	–	6.96 (5.02-9.66)	–

PMM = Predicted Mean Mortality. EMM = Estimated Marginal Means of mortality. IRR = Incidence Rate Ratios. SD = Standard Deviation. CI = Confidential Interval.

Table S7. Multivariable negative binomial regression analysis of factors associated with monthly cetacean mortality in the Lower Gulf of Thailand.

Variable	IRR (95% CI)		Adjusted mortality (deaths/month, 95% CI)	P-value
**Sea surface temperature (per 1 °C increase)**	0.78 (0.53–1.16)	Low	12.86 (9.47–17.45)	0.227
**χ^2 = 1.44, df = 1, P = 0.23**		High	6.39 (4.53–9.00)	
**Wind speed (per 1 km/h increase)**	1.03 (0.82–1.29)	Low	8.87 (6.42–12.26)	0.796
**χ^2 = 0.07, df = 1, P = 0.80**		High	9.16 (6.60–12.70)	
**Fishery production (per 1000 tons increase)**	1.00 (0.99–1.00)	Low	9.15 (6.78–12.35)	0.654
**χ^2 = 0.20, df = 1, P = 0.65**		High	9.10 (6.75–12.29)	

PMM = Predicted Mean Mortality. EMM = Estimated Marginal Means of mortality. IRR = Incidence Rate Ratios. CI = Confidential Interval.
